# Increased Calbindin D28k Expression via Long-Term Alternate-Day Fasting Does Not Protect against Ischemia-Reperfusion Injury: A Focus on Delayed Neuronal Death, Gliosis and Immunoglobulin G Leakage

**DOI:** 10.3390/ijms22020644

**Published:** 2021-01-11

**Authors:** Hyejin Sim, Tae-Kyeong Lee, Yeon Ho Yoo, Ji Hyeon Ahn, Dae Won Kim, Bora Kim, Jae-Chul Lee, Joon Ha Park, Soon-Sung Lim, Jung-Seok Park, Il Jun Kang, Young-Myeong Kim, Myoung Cheol Shin, Jun Hwi Cho, Yoonsoo Park, Moo-Ho Won

**Affiliations:** 1 Department of Neurobiology, School of Medicine, Kangwon National University, Chuncheon, Gangwon 24341, Korea; janny20@naver.com (H.S.); jh-ahn@ysu.ac.kr (J.H.A.); nbrkim17@gmail.com (B.K.); anajclee@kangwon.ac.kr (J.-C.L.); 2 Department of Biomedical Science and Research Institute for Bioscience and Biotechnology, Hallym University, Chuncheon, Gangwon 24252, Korea; tk-lee@hallym.ac.kr; 3 Department of Emergency Medicine, Institute of Medical Sciences, Kangwon National University Hospital, School of Medicine, Kangwon National University, Chuncheon, Gangwon 24289, Korea; yhounho@gmail.com (Y.H.Y.); dr10126@naver.com (M.C.S.); cjhemd@kangwon.ac.kr (J.H.C.); 4 Department of Physical Therapy, College of Health Science, Youngsan University, Yangsan, Gyeongnam 50510, Korea; 5 Department of Biochemistry and Molecular Biology, Research Institute of Oral Sciences, College of Dentis try, Gangnung-Wonju National University, Gangneung, Gangwon 25457, Korea; kimdw@gwnu.ac.kr; 6 Department of Anatomy, College of Korean Medicine, Dongguk University, Gyeongju, Gyeongbuk 38066, Korea; jh-park@dongguk.ac.kr; 7 Department of Food Science and Nutrition, College of Natural Sciences, Hallym University, Chuncheon, Gangwon 24252, Korea; limss@hallym.ac.kr (S.-S.L.); ijkang@hallym.ac.kr (I.J.K.); 8 Department of Physical Education, College of Natural Science, Hallym University, Chuncheon, Gangwon 24252, Korea; 41920@hallym.ac.kr; 9 Department of Molecular and Cellular Biochemistry, School of Medicine, Kangwon National University, Chuncheon, Gangwon 24341, Korea; ymkim@kangwon.ac.kr

**Keywords:** intermittent fasting, ischemia-reperfusion, calcium binding protein, hippocampal CA1 region, pyramidal neurons

## Abstract

Calbindin-D28k (CB), a calcium-binding protein, mediates diverse neuronal functions. In this study, adult gerbils were fed a normal diet (ND) or exposed to intermittent fasting (IF) for three months, and were randomly assigned to sham or ischemia operated groups. Ischemic injury was induced by transient forebrain ischemia for 5 min. Short-term memory was examined via passive avoidance test. CB expression was investigated in the Cornu Ammonis 1 (CA1) region of the hippocampus via western blot analysis and immunohistochemistry. Finally, histological analysis was used to assess neuroprotection and gliosis (microgliosis and astrogliosis) in the CA1 region. Short-term memory did not vary significantly between ischemic gerbils with IF and those exposed to ND. CB expression was increased significantly in the CA1 pyramidal neurons of ischemic gerbils with IF compared with that of gerbils fed ND. However, the CB expression was significantly decreased in ischemic gerbils with IF, similarly to that of ischemic gerbils exposed to ND. The CA1 pyramidal neurons were not protected from ischemic injury in both groups, and gliosis (astrogliosis and microgliosis) was gradually increased with time after ischemia. In addition, immunoglobulin G was leaked into the CA1 parenchyma from blood vessels and gradually increased with time after ischemic insult in both groups. Taken together, our study suggests that IF for three months increases CB expression in hippocampal CA1 pyramidal neurons; however, the CA1 pyramidal neurons are not protected from transient forebrain ischemia. This failure in neuroprotection may be attributed to disruption of the blood–brain barrier, which triggers gliosis after ischemic insults.

## 1. Introduction

Intermittent fasting (IF) entails alternate cycles of feeding and fasting to induce energy and dietary restriction [1]. A few studies using rodent models of focal cerebral ischemia have demonstrated that IF increases resistance to ischemia/reperfusion injury in rodent brains [1,2,3,4]. In these studies, IF attenuates tissue damage (infarction) and neurological deficit following focal brain ischemia, demonstrating that IF acts as a mild metabolic stressor in neurons or glial cells and effectively upregulates the expression of several neuroprotective antioxidant enzymes, inflammatory mediators, and calcium-binding proteins. However, recent studies suggest that IF in a gerbil model of 5-minute transient forebrain ischemia (TFI) does not protect neurons [5].

Irreversible neuronal death in the gerbil model occurs specifically in vulnerable subregions of the brain, including the striatum, neocortex and hippocampus [6,7]. In particular, pyramidal neurons in the hippocampal cornu ammonis 1 (CA1) are vulnerable to ischemic insults and are prone to die several days after 5-minute episodes of TFI, and this selective neuronal death is designated as “delayed neuronal death” [8,9]. It is well known that glial cells (microglia and astrocytes) proliferate with hypertrophied cell bodies, and this “reactive gliosis (microgliosis and astrogliosis)”, which occurs early after 5-minute TFI, is gradually enhanced with time until delayed neuronal death occurs [9,10]. In addition, gliosis in CNS insults is initiated after the disruption of the blood–brain barrier (BBB), allowing non-CNS molecules including blood and serum components to enter the brain parenchyma [11,12,13,14].

Among neuroprotective factors against brain insults, calbindin-D28k (CB), one of the major calcium-binding and buffering proteins, maintains intracellular calcium homeostasis and plays a critical role in protecting neurons against calcium-mediated neurotoxicity [15,16,17,18]. Ca^2+^ is an important intracellular messenger controlling cellular differentiation, growth, membrane excitability and synaptic activity [19]. Ischemic insults lead to an excessive intracellular influx of Ca^2+^ [1], which results in neuronal death [20]. In addition, CB-containing neurons play an important role in learning, memory, cognitive function, and long-term potentiation [21,22]. Hippocampal CB expression may influence memory function via neuronal calcium homeostasis [23].

Based on ongoing studies, IF and/or CB exhibit favorable effects on memory and cognitive function. To date, however, the mechanism of the IF-induced modulation of memory and cognitive function, and CB expression, is unclear. To the best of our knowledge, studies have yet to analyze these IF-induced effects on gerbil brains exposed to 5-minute TFI. Therefore, we investigated whether IF influenced passive avoidance tests, which are used to determine learning and short-term memory, CB expression, neuronal survival, reactive gliosis and BBB leakage (disruption) in the hippocampal CA1region, in which the pyramidal neurons are very vulnerable to transient ischemia in gerbils with 3-month IF.

## 2. Results

We previously presented a change in body weight caused by IF and normal diet for three months, showing that no significant difference in body weights was detected between IF-subjected gerbils and normal-dieted gerbils (data not shown) [11,24].

### 2.1. Passive Avoidance Test (PAT)

**Figure 1:**
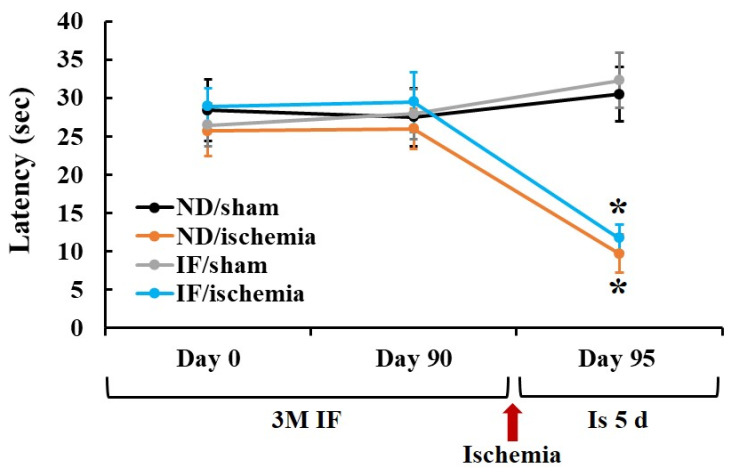
Short-term memory tests using passive avoidance test in the ND/sham, IF/sham, ND/ischemia and IF/ischemia groups. The latency time before and after 3-month IF in both sham groups is not altered. In addition, there is no difference in latency time at 5 days post-ischemia between the ND/ischemia and IF/ischemia groups (*n* = 7 per group; * *p* < 0.05, vs. ND/sham group by post hoc Tukey’s test). Bars indicate means ± SEM. IF, intermittent fasting; ND, normal diet.

PAT was performed to examine the effect of IF on learning and memory following TFI (Figure 1). No significant difference was shown in the latency time after 3 months of IF between the normal diet (ND)/sham and the IF/sham groups. At 5 days after ischemia, a significant reduction (*p* < 0.001) in the latency time was shown in both ND/ischemia and IF/ischemia groups compared to that in the ND/sham group, showing that the latency time in both of the groups was similar. These results demonstrate that the short-term memory function in the IF/ischemia group was not different from that in the ND/ischemia group.

### 2.2. CB Protein Levels

**Figure 2:**
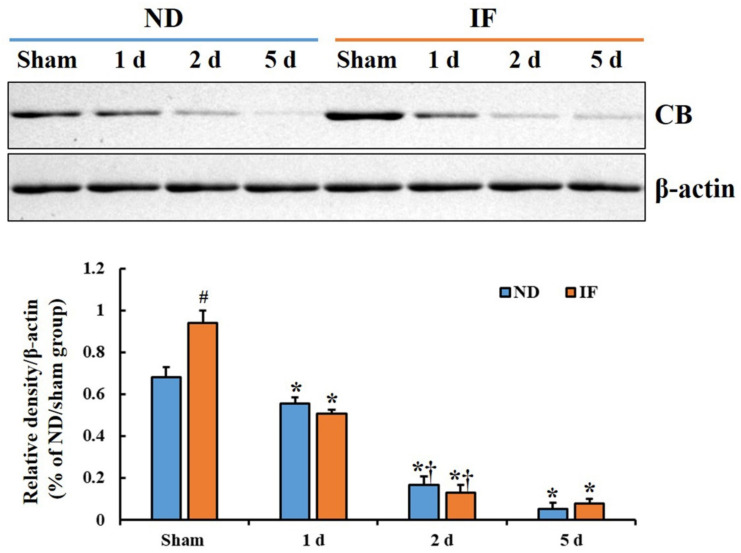
Representative blot images and quantitative analysis of CB protein in the hippocampal CA1 region of the ND/sham, IF/sham, ND/ischemia and IF/ischemia groups at sham, 1 day, 2 days, and 5 days after ischemia (*n* = 5 at each point in time in each group, * *p* < 0.05 vs. sham group, ^#^ *p* < 0.05 vs. corresponding time point group of ND group, ^†^ *p* < 0.05 vs. pre-time point group of each group by post hoc Tukey’s test). Bars indicate means ± SEM. IF, intermittent fasting; ND, normal diet.

The CB protein level in the hippocampal CA1 of the ND/ischemia group was significantly decreased 1 day after ischemia and gradually decreased until 5 days after ischemia (Figure 2). In the IF/sham group, the CB protein level was significantly increased (*p* < 0.001) after 3-month IF compared with that in the ND/sham group (Figure 2). However, the CB protein level in this group was significantly reduced (*p* < 0.001) from 1 day to 5 days after ischemia, showing that the change pattern after ischemia was similar to that of the ND/ischemia group (Figure 2).

### 2.3. CB Immunoreactivity

**Figure 3:**
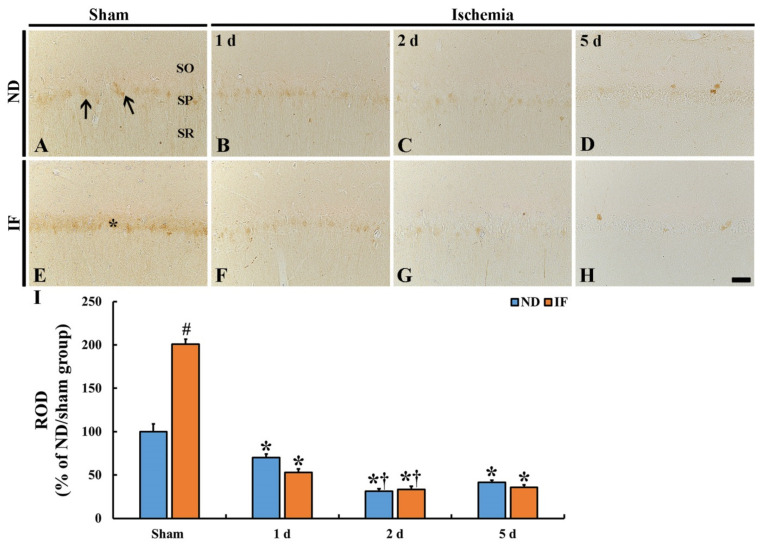
Calbindin D28K (CB) immunohistochemistry in the CA1 region of the ND (upper columns) and the IF (lower columns) groups at sham (**A**,**E**), 1 day (**B**,**F**), 2 days (**C**,**G**) and 5 days (**D**,**H**) after ischemia CB immunoreactivity is shown in CA1 pyramidal neurons (arrows). CB immunoreactivity in the CA1 pyramidal neurons (asterisk) in the IF/sham group is significantly higher than that in the ND/sham group. However, the CB immunoreactivities in both of the groups decreases gradually and similarly with time after ischemia. Scale bar = 50 µm. (**I**) relative optical density (ROD) of CB immunoreactivity as percent value in the CA1 region (*n* = 7 in each group, * *p* < 0.05 vs. ND/sham group, ^#^ *p* < 0.05 vs. corresponding time point group of ND group, ^†^ *p* < 0.05 vs. pre-time point group of each group by post hoc Tukey’s test). Bars indicate the means ± SEM. IF, intermittent fasting; ND, normal diet; SO, stratum oriens; SP, stratum pyramidale; SR, stratum radiatum.

As shown in Figure 3A, CB immunoreactivity in the ND/sham group was shown in the stratum pyramidale (SP) of the hippocampal CA1. CB immunoreactivity in the ND/ischemia group was significantly and gradually decreased (*p* < 0.001) from 1 day to 5 days after ischemia, showing that relative optical density (ROD) was 70% at 1 day, 31% at 2 days, and 41% at 5 days post-ischemia compared with that in the ND/sham group (Figure 3B–D,I).

The CB immunoreactivity in the CA1 of the IF/sham group had almost doubled (about 200% of the ND/sham group) compared with that in the ND/sham group (Figure 3E). In the IF/ischemia group, the CB immunoreactivity in the SP of the CA1 region was also significantly decreased (*p* < 0.001) with time after ischemia (53% at 1 day, 33% at 2 days, and 36% at 5 days post-ischemia when compared with that in the ND/sham group) (Figure 3F–I), showing that the ROD pattern was similar to that found in the ND/ischemia group (Figure 3I). These finding indicate that IF for 3 months increased CB expression; however, the IF did not maintain CB expression in the CA1 after transient ischemia.

### 2.4. Neuroprotection

#### 2.4.1. Cresyl Violet (CV) Positive Cells

**Figure 4:**
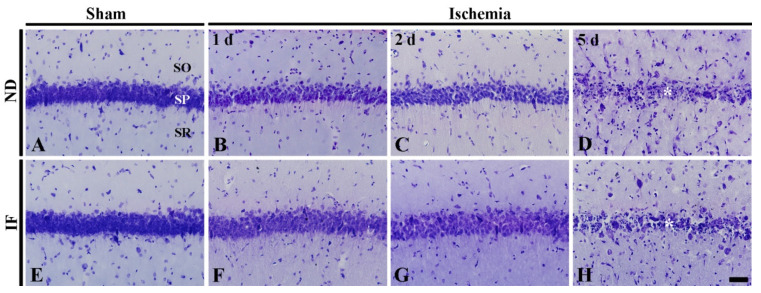
Cresyl violet (CV) staining in the CA1 region of the ND (upper column) and IF (lower column) groups at sham (**A**,**E**), 1 day (**B**,**F**), 2 days (**C**,**G**) and 5 days (**D**,**H**) after ischemia. Numerous CV-positive cells are found in stratum pyramidale (SP). CV stainability of cells in the SP is gradually decreased after ischemia in both of the ND/ischemia and IF/ischemia groups, and, at 5 days after ischemia, CV-positive cells in the SP of the groups are damaged (asterisks). Scale bar = 50 µm. IF, intermittent fasting; ND, normal diet; SO, stratum oriens; SR, stratum radiatum.

CV staining was performed to investigate the distribution and morphology of cells located in the hippocampal CA1 region (Figure 4). In the ND/sham group, numerous CV-positive cells located in the stratum pyramidale were CA1 pyramidal cells (Figure 4A). In the ND/ischemia group, the patterns of CV-positive CA1 pyramidal cells were not altered at 1 day and 2 days after ischemia, although the CV stainability was decreased with time after ischemia (Figure 4B,C). At 5 days after ischemia, most of the CV-positive pyramidal cells were damaged at 5 days post-ischemia (Figure 4D).

In the IF/sham group, the distribution of CV-positive pyramidal cells was not different from that in the ND/ischemia group (Figure 4E). In the IF/ischemia group, the change pattern of CV-positive pyramidal cells was similar to that found in the ND/ischemia group (Figure 4F–H).

#### 2.4.2. Neuronal Nuclear Antigen (NeuN) Immunoreactive Neurons

**Figure 5:**
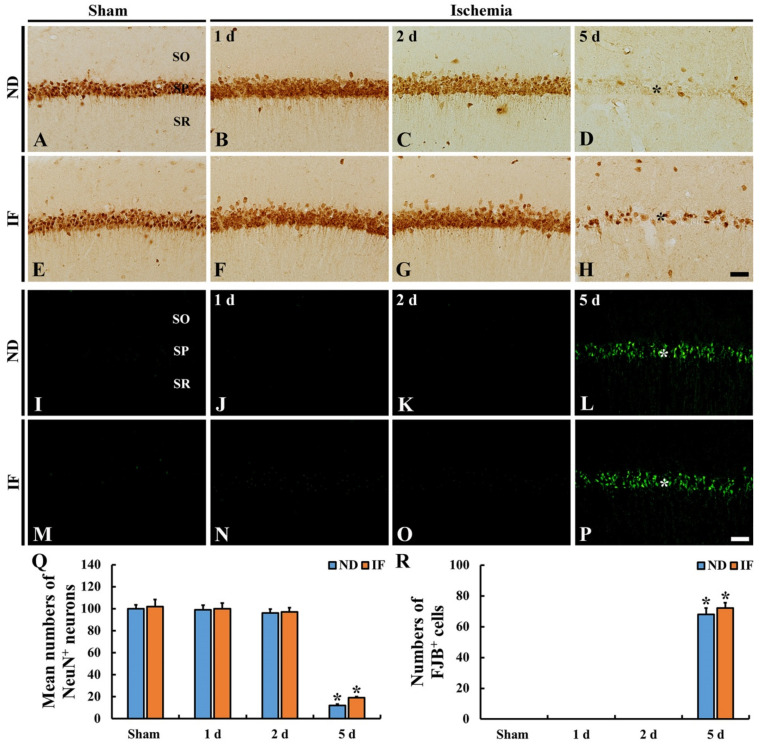
Neuronal nuclei antigen (NeuN) immunohistochemistry (**A**–**H**) and fluoro-Jade B (FJB) fluorescence staining (**I**–**P**) in the CA1 region of the ND and IF groups at sham (**A**,**E**,**I**,**M**), 1 day (**B**,**F**,**J**,**N**), 2 days (**C**,**G**,**K**,**O**) and 5 days (**D**,**H**,**L**,**P**) after ischemia. The NeuN immunoreactive pyramidal neurons in both of the groups show immunoreactivity until 2 days post-ischemia; however, at 5 days post-ischemia, few NeuN immunoreactive pyramidal neurons (asterisk) are shown in both of the groups. In addition, in both of the groups, numerous FJB-positive neurons are shown in the stratum pyramidale (SP) at 5 days post-ischemia. Scale bar = 50 µm. (**Q**,**R**) Mean numbers of NeuN immunoreactive (**Q**) and FJB-positive (**R**) cells in 250 μm^2^ at the center of the CA1 region (*n* = 7 in each group, * *p* < 0.05 vs. ND/sham group by post hoc Tukey’s test). Bars indicate the means ± SEM. IF, intermittent fasting; ND, normal diet; SO, stratum oriens; SR, stratum radiatum.

NeuN (a marker for neurons) immunoreactivity was observed in CA1 pyramidal neurons of the ND/sham groups (Figure 5A). In the ND/ischemia group, NeuN-immunoreactive neurons were not significantly altered (*p* < 0.001) in number at 1 day and 2 days (about 98% and 95% of the ND/sham group, respectively) after ischemia (Figure 5B,C,Q), showing that the intensity of NeuN immunoreactivity at 2 days post-ischemia was weaker than that in the ND/sham group (Figure 5C). At 5 days post-ischemia, however, the numbers of NeuN immunoreactive CA1 pyramidal neurons were dramatically reduced (*p* < 0.001) (about 12% of the ND/sham group) (Figure 5D,Q).

In the IF/sham group, the numbers and NeuN immunoreactivity in the CA1 pyramidal neurons did not differ from those in the ND/sham group (Figure 5E,Q). In the IF/ischemia group, the number of NeuN-immunoreactive CA1 pyramidal neurons at 1 day and 2 days post-ischemia was similar to that found in the ND/ischemia group (Figure 5F,G,Q), showing that, at 2 days post-ischemia, NeuN immunoreactivity was still strong (Figure 5G). At 5 days after ischemia, the number of NeuN immunoreactive CA1 pyramidal neurons was also markedly decreased (*p* < 0.001) (about 19% of the ND/sham group) (Figure 5H,Q).

#### 2.4.3. Fluoro-Jade B (FJB)-Positive Cells

FJB (a high-affinity fluorescent marker for neuronal degradation)-positive cells were not observed in the ND/sham or IF/sham groups (Figure 5I,J). After ischemia in both of the groups, FJB-positive cells were not detected until 2 days after ischemia (Figure 5J,K,N,O,R). At 5 days following ischemia, FJB-positive cells were detected in both of the groups (Figure 5L,P), showing that there was no significant difference in the numbers of FJB-positive cells between the ND/ischemia and IF/ischemia groups (Figure 5R).

### 2.5. Microgliosis

**Figure 6:**
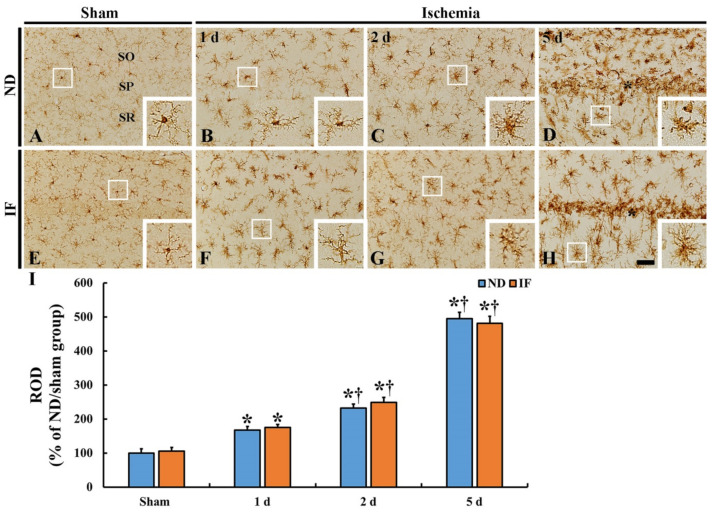
Ionized calcium-binding adapter molecule 1 (Iba-1) immunohistochemistry in the CA1 region of the ND (upper column) and IF (lower column) groups at sham (**A**,**E**), 1 day (**B**,**F**), 2 days (**C**,**G**) and 5 days (**D**,**H**) after ischemia. In both of the groups, Iba-1 immunoreactivity is significantly increased with time following ischemia, showing no difference in the immunoreactivity between the two groups. Note that Iba-1 immunoreactive cells are numerous near the stratum pyramidale (SP) (asterisk), in which pyramidal neurons are dead. Scale bar = 50 µm. (**I**) Relative optical density (ROD) of Iba-1 immunoreactivity as percent values in the CA1 region (*n* = 7 in each group, * *p* < 0.05 vs. ND/sham group, ^†^ *p* < 0.05 vs. pre-time point group of each group by post hoc Tukey’s test). Bars indicate the means ± SEM. IF, intermittent fasting; ND, normal diet; SO, stratum oriens; SR, stratum radiatum.

Immunohistochemical staining for ionized calcium-binding adapter molecule 1 (Iba-1), a marker for microglia, was done to observe microglia activation in the CA1 region in the ND and IF groups with or without ischemia (Figure 6). In the ND/sham (Figure 6A) and IF/sham (Figure 6E) groups, there was no difference in the distribution of Iba-1 immunoreactive microglia between the two groups. Namely, they were evenly scattered in all layers of the CA1 region and identified as a resting form of microglia (Figure 6A,E). After ischemia in the two groups, the change patterns of the Iba-1 immunoreactive microglia were similar (Figure 6B–D,F–H). With time after ischemia, their cytoplasm was enlarged with thickened processes as the active form. In addition, no significant difference in the ROD of Iba-1 immunoreactivity was observed between the two groups. The ROD in the ND/ischemia group was gradually increased (167% at 1 day, 231% at 2 days, and 494% at 5 days compared to that of the ND/sham group) (Figure 6I). In the IF/ischemia group, the ROD was 175% at 1 day, 248% at 2 days, and 481% at 5 days, compared to that of the ND/sham group (Figure 6I).

### 2.6. Astrogliosis

**Figure 7:**
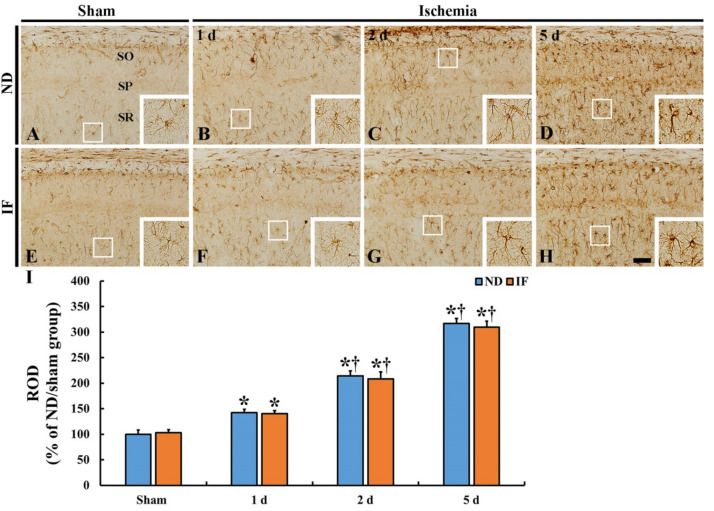
Glial fibrillary acidic protein (GFAP) immunohistochemistry in the CA1 region of the ND (upper column) and IF (lower column) groups at sham (**A**,**E**), 1 day (**B**,**F**), 2 days (**C**,**G**) and 5 days (**D**,**H**) after ischemia. In both of the groups, the GFAP immunoreactivity is significantly increased with time following ischemia, showing no difference in the immunoreactivity between the two groups. Scale bar = 50 µm. (**I**) Relative optical density (ROD) of GFAP immunoreactivity as percent values in the CA1 region (*n* = 7 in each group, * *p* < 0.05 vs. ND/sham group, ^†^ *p* < 0.05 vs. pre-time point group of each group by post hoc Tukey’s test). Bars indicate the means ± SEM. IF, intermittent fasting; ND, normal diet; SO, stratum oriens; SP, stratum pyramidale; SR, stratum radiatum.

Immunohistochemical staining for glial fibrillary acidic protein (GFAP), a marker for astrocytes, was carried out in order to investigate the activation of astrocytes in the CA1 region in the ND and IF groups with or without ischemia (Figure 7). GFAP immunoreactive astrocytes, as the resting form, had fine cellular processes in both ND/sham (Figure 7A) and IF/sham groups (Figure 7E). On the other hand, after ischemia, the pattern of changes in the GFAP immunoreactive astrocytes was similar between the ND and IF groups (Figure 7B–D,F–H). With time after ischemia, GFAP immunoreactive astrocytes became hypertrophied, and their cellular processes became thickened in both of the ND and IF/ischemia groups (Figure 7B–D,F–H). ROD in the ND/ischemia group was gradually increased (142% at 1 day, 214% at 2 days, and 316% at 5 days compared to that of the ND/sham group) after ischemia (Figure 7I). In the IF/ischemia group, the ROD was 140% at 1 day, 207% at 2 days, and 309% at 5 days compared to that of the ND/sham group (Figure 7I).

### 2.7. Immunoglobulin G (IgG) Immunoreactivity

**Figure 8:**
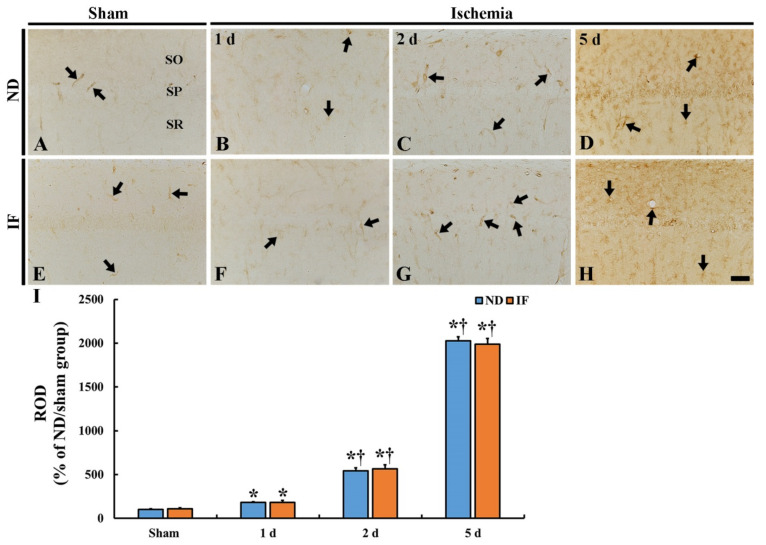
Immunoglobulin G (IgG) immunohistochemistry in the CA1 region of the ND (upper column) and IF (lower column) groups at sham (**A**,**E**), 1 day (**B**,**F**), 2 days (**C**,**G**) and 5 days (**D**,**H**) after ischemia. In the two groups, IgG immunoreactivity is significantly increased with time after ischemia, showing that there is no difference in the immunoreactivity between the two groups. Scale bar = 50 µm. (**I**) Relative optical density (ROD) of IgG immunoreactivity as percent values in the CA1 region (*n* = 7 in each group, * *p* < 0.05 vs. ND/sham group, ^†^ *p* < 0.05 vs. pre-time point group of each group by post hoc Tukey’s test). Bars indicate the means ± SEM. IF, intermittent fasting; ND, normal diet; SO, stratum oriens; SP, stratum pyramidale; SR, stratum radiatum.

To investigate BBB leakage due to the disruption of the BBB after ischemia, immunohistochemistry for IgG was conducted in the CA1 region in the ND and IF gerbils with or without ischemia (Figure 7). IgG immunoreactivity was fundamentally shown inside of the blood vessels in both the ND/sham (Figure 8A) and IF/sham groups (Figure 8E) (arrows). However, in both the ND and IF groups with ischemia, IgG immunoreactivity was shown near or outside the blood vessels (arrows), and was significantly enhanced (*p* < 0.001) (ND and IF, 183% and 181%, respectively) at 1 day, and a more significant increase (544% and 564%, respectively) was detected at 2 days compared to that in the ND/sham group (Figure 8B,C,F,G,I). At 5 days after ischemia, the IgG immunoreactivity was dramatically increased (ND and IF, 2027% and 1987%, respectively) in both the ND and IF/ischemia groups compared to that in the ND/sham group (Figure 8D,H,I).

## 3. Discussion

The three major mechanisms underlying the neuronal death induced by transient global brain or forebrain ischemia include the following: (1) oxidative stress induced by overproduction of reactive oxygen species (ROS), (2) inflammatory response by pro-inflammatory cytokines and immune cells, and (3) glutamate-induced excitotoxicity [25,26,27]. Accordingly, many studies have reported the factors underlying neuroprotection against TFI. The enhanced expression of antioxidant enzymes contributes to ROS scavenging, and the elevation of anti-inflammatory cytokines triggers inflammation in ischemic brains by suppressing the expression of pro-inflammatory cytokines, and the increased levels of calcium-binding proteins attenuate excitotoxicity by buffering glutamate influx [28,29]. Therefore, we investigated whether an IF-mediated increase in CB expression influenced neuronal survival, reactive gliosis and BBB leakage (disruption) in the hippocampal CA1 region.

The hippocampus is critical for memory and cognitive function [30]. IF increases the thickness of the hippocampal CA1 region in mice, suggesting enhanced learning and memory [31]. In addition, alternate-day IF has been shown to improve memory, sensory and motor skills [32,33,34]. In this study, we evaluated short-term memory after TFI with or without 3-month IF in gerbils, and found no significant difference in function between groups with ND/ischemia and IF/ischemia as well as groups exposed to ND/sham and IF/sham. These findings differ from those reported in foregoing studies. Based on our current study, the long-term (3 months) exposure of gerbils to alternate-day IF does not increase short-term memory. In this regard, we evaluated the expression of CB in cognitive and memory function [35], neuroprotection and reactive gliosis (microgliosis and astrogliosis) in the hippocampal CA1 region of the four groups (ND/sham, ND/ischemia, IF/sham and IF/ischemia) to determine the factors underlying the lack of short-term memory enhancement following IF.

The CB levels are decreased and the Ca^2+^ influx is increased in brains with aging, which influences memory and cognitive function decline [36,37,38]. Based on these studies, the increased CB expression enhances memory and cognitive function. In our current study, we found that CB expression was significantly increased in the IF/sham group after 3 months of IF compared with that of the ND/sham group; however, based on PAT, the learning and memory performance following 3-month IF was not enhanced. We also found that CB expression following ischemia declined gradually with time after ischemia in the IF/ischemia group, similar to that of the ND/sham group. This result indicates that the 3-month IF in gerbils does not sustain CB expression after ischemic insult despite the increased CB expression in CA1 pyramidal neurons following 3-month IF. Based on the findings of altered CB in the IF/ischemia group, we analyzed the degrees of neuroprotection and reactive gliosis in the IF/ischemia group. The findings indicate that the death (loss) of CA1 pyramidal neurons and reactive gliosis in the IF/ischemia group was similar to that of the ND/ischemia group, suggesting that IF for 3 months in gerbils does not protect against TFI.

Dietary restriction or caloric restriction in animal models of focal or global ischemia significantly reduces neurological damage [4,39,40,41,42]. In addition, a few studies have demonstrated that IF induces neuroprotective effects by attenuating cellular dysfunction, degeneration and death in the brain after experimental focal brain ischemia [43], in contrast to the transient forebrain ischemia induced in our study. We recently reported that IF for 3 months in a gerbil model of TFI increased the expression of endogenous antioxidant enzymes (SOD1, SOD2, and catalase) without protecting the hippocampal CA1 pyramidal neurons from ischemic injury [5]. Similarly, in our current study, the increased CB levels following IF failed to protect the CA1 pyramidal neurons in the IF/ischemia group against TFI.

Several studies have reported that CB levels play a key role in neuroprotection or neuronal survival in the brains of patients with neurodegenerative disorder or injury [44,45,46,47]. In the case of focal brain ischemia involving rabbits, the increased CB protects the brain tissue against focal ischemia, suggesting that the increased CB blocks intracellular calcium entry and protects the brain against focal ischemia [48,49]. In contrast, it has been reported that CB fails to protect hippocampal neurons from transient global brain ischemia in spite of its cytoplasmic calcium-buffering properties observed in CB knockout mice [44].

Ischemic insults trigger reactive gliosis in the brain [9,10]. Attenuated reactive gliosis in ischemic brains is a measure of neuroprotection against ischemic insults [10,50,51]. Our results showed the development of microgliosis and astrogliosis, which was enhanced with time in the CA1 region of both the ND and IF groups after 5 min of TFI, although the differences between the two groups were not statistically different. This finding indicates that ischemia-induced gliosis, which leads to neuronal damage/death, cannot be prevented by 3-month IF. Some studies have reported that gliosis following brain insults begins after the disruption of the BBB, which allows blood and serum components to enter the ischemic brain parenchyma [11,12,13,14]. We recently reported that 1-, 2- and 3-month IF did not prevent BBB leakage in the gerbil hippocampal CA1 region 5 days after 5-minute TFI [11]. In addition, we found that interleukin-13 (an anti-inflammatory cytokine), which plays a beneficial role in ischemic injury, was significantly increased in the ischemic CA1 region, but there was no neuroprotective effect against TFI [11]. Taken together, the BBB leakage in ischemic brains may fail to protect neurons from ischemic insults.

Our present study was performed using gerbils with a mean lifespan of 110 weeks in males and 139 weeks in females [52]. In this regard, the 3-month IF period in gerbils may translate to approximately 10 years in human beings. The 3-month duration should be adequate for gerbils to adapt to the new dietary protocol, which may explain the lack of neuroprotective effects following transient ischemia with 3-month IF. This finding suggests that the effects of long-term IF on brains may differ from those of dietary or caloric restriction following various brain injuries, in particular ischemic insults.

Ischemic stroke is a representative senile disease, and several studies have investigated various outcomes after ischemic insults using aged animals [50,51,53,54]. The current data, however, relate to adult animals, suggesting the need for additional and identical studies involving aged animals.

In brief, IF for 3 months increased the production of CB in hippocampal CA1 pyramidal neurons in gerbils. However, the 3-month IF did not prevent BBB disruption or reactive gliosis, which might lead to the death of CA1 pyramidal neurons after 5 min of TFI. Further studies with diverse and modified IF regimens are necessary to determine the neuroprotective effects after TFI, and to establish the type of IF protocol that prevents or protects against ischemic brain injury.

## 4. Materials and Methods

### 4.1. Experimental Animals

Male Mongolian gerbils (*Meriones unguiculatus*) at six months of age (body weight, 75 ± 5 g) were obtained from the Experimental Animal Center (Kangwon University, Chuncheon, Gangwon, Republic of Korea). They had been maintained at a constant temperature (23 ± 2 °C) and humidity (50 ± 5%) with a 12 h light/dark cycle. The care and handling of animals in this research complied with the “Guidelines of the Current International Laws and Policies” described in the “Guide for the Care and Use of Laboratory Animals” (The National Academies Press, 8th Ed., 2011). In addition, the protocol of this experiment was approved (approval number: KW-200113-1) by the committee of Institutional Animal Care and Use Committee at Kangwon National University.

### 4.2. IF and Experimental Groups

For IF, gerbils were allowed free access to ND every other day and no food on alternate days (24 h fasting and 24 h feeding), and IF was applied for three months according to the published method [3,32,55]. During the feeding period, food intake in all gerbils was controlled daily (10 g/day), and body weight was monitored every week.

For experimental groups, 96 male gerbils were used and randomly divided into four groups, as follows: (1) ND/sham group (*n* = 12)—gerbils were fed ND and received sham ischemia operation; (2) ND/ischemia group (*n* = 36)—animals were fed ND and received ischemia; (3) IF/sham group (*n* = 12)—gerbils had IF for three months and received sham operation; (4) IF/ischemia group (*n* = 36)—animals had IF and received ischemia. The animals in the ischemia group were sacrificed at 1 day, 2 days, and 5 days after ischemia to investigate the effects of IF on CB expression and neuroprotection following transient global forebrain ischemia. To decrease the number of gerbils, gerbils in the sham group were sacrificed only at 5 days after the sham ischemia operation.

### 4.3. Induction of Transient Forebrain Ischemia

As we previously described [5], all gerbils were anesthetized with a mixture of 2.5% isoflurane from Baxtor (Deerfield, IL, USA) in 67% nitrous oxide and 33% oxygen. Under anesthesia, the gerbils received an incision on the neck to find both common carotid arteries, and the arteries were occluded by non-traumatic aneurysm clips for 5 min and then re-perfused. The body temperature of the gerbils was controlled at normothermia (37 ± 0.5 °C) using a thermometric blanket during the surgery, monitoring the temperature using a rectal temperature probe (TR-100) (Fine Science Tools, Foster City, CA, USA). All animals were fed ND after the surgical procedure. The gerbils included in the sham group underwent identical surgical procedures without the ligation of common carotid arteries. Thereafter, the gerbils were kept in thermal incubators (temperature, 23 °C; humidity, 60%) to maintain body temperature at the normothermic level until they were euthanized. Until the gerbils were sacrificed, they were continually fed ND or IF.

### 4.4. PAT

Learning and memory was assayed through PAT according to the modification of a published method [56]. Shortly, the Gemini Avoidance System (GEM 392) (San Diego Instruments, San Diego, CA, USA) was used for this PAT, which consisted of two rooms (light and dark) with a grid floor. First, training was performed one day before IF, 29 days after IF and 4 days after ischemia, as follows. For the training, each gerbil was allowed to explore the environment of the two rooms for one min, while the grid floor was opened. After the exploration, the gerbil was permitted to enter the dark room when a light in the light room was turned on, while the floor was given an inescapable foot-shock (0.5 mA for 5 s). A substantive PAT test was done 24 h after the training. Each trained gerbil was put in the dark room, the light of the light room was turned on, and the floor was opened. Thereafter, we recorded the latency time, which is the time to enter the dark room, within 180 s.

### 4.5. Western Blotting

To examine changes of CB protein level in the hippocampal CA1, 40 gerbils (*n* = 5 at sham, 1, 2 and 5 days post-ischemia in each group) were used for the blotting according to a previously published method [57]. Shortly, at each point in time, five gerbils in each group were deeply anesthetized by an intraperitoneal injection of pentobarbital sodium (60 mg/kg) from JW Pharm (Seoul, South Korea), and their hippocampal CA1 tissues were collected. The tissues were lysed with RIPA buffer (Santa Cruz, CA, USA) and homogenized with an ultrasonic homogenizer for 5 min. These homogenates were then centrifuged at 12,000 rpm for 20 min at 4 °C, and the supernatants were collected. Next, protein concentrations were measured with a bicinchoninic acid kit from Thermo Fisher Scientific (Waltham, MA, USA). In detail, the proteins were separated by 10% sodium dodecyl sulfate–polyacrylamide gel and transferred to nitrocellulose membranes (Pall Corp., Pittsburgh, PA, USA). These membranes were blocked with 5% non-fat milk (in Tris-buffered saline/Tween, TBST) for 60 min on a shaker at room temperature and then incubated in primary antibodies (rabbit anti-CB) (diluted 1:1000) (Cell signaling technology, Danvers, MA, USA) and rabbit anti-β-actin (42 kDa) (1:2000, Sigma-Aldrich, St. Louis, MO, USA) overnight at 4 °C. The membranes were washed three times with TBST and incubated with peroxidase conjugated anti-rabbit IgG (1:4000, Santa Cruz, CA, USA) for 1 h at room temperature. After washing them with TBST, they were visualized by horseradish peroxidase (Millipore, Billerica, MA, USA). The band intensities were analyzed using ImageJ (ver. 1.52v, National Institutes of Health, Bethesda, MD, USA).

### 4.6. Preparation of Histological Sections

At each point in time after TFI, seven gerbils in each group were deeply anesthetized by intraperitoneal injection of 60 mg/kg of pentobarbital sodium (JW Pharm. Co., Ltd., Seoul, Korea) at designated times (1, 2 and 5 days after ischemia) following IF. Under anesthesia, the gerbils were transcardially washed with 0.1 M phosphate buffered saline (PBS) (pH 7.4) and fixed with 4% paraformaldehyde solution (in 0.1 M PB, pH 7.4). After fixation, their brains were removed, and cryoprotected in 30% sucrose solution. Finally, brain tissues containing the hippocampus were serially sectioned into coronal sections (30 μm thickness) in a cryostat of Leica (Wetzlar, Germany) and kept in plates containing PBS.

### 4.7. Immunohistochemistry

We used the following antibodies: rabbit anti-CB (diluted 1:1000; Cell signaling technology, Danvers, MA, USA), NeuN (diluted 1:800; Abcam, Cambridge, MA, USA), Iba-1 (1:800, Wako, Japan) and GFAP (1:1000, Chemicon, Temecula, CA, USA). For immunohistochemistry for each antibody, we carried it out according to our published method [38]. Shortly, the sections obtained at designated times after ischemia were incubated in 0.3% hydrogen peroxide (H_2_O_2_) solution for 30 min, followed by 10% normal goat serum solution (in 0.05 M PBS, pH 7.4) for 30 min. These sections were reacted with each antibody for 18 h at 4 °C. Continuously, they were exposed to biotinylated anti-rabbit IgG (diluted 1:200, Vector, Burlingame, CA, USA) and streptavidin peroxidase complex (diluted 1:200, Vector, Burlingame, CA, USA) for 2 h at room temperature, respectively. Finally, the reacted sections were visualized by incubating in a 0.05% solution of 3, 3′-diaminobenzidine tetrahydrochloride (DAB) (in 0.1 M Tris–HCl buffer, pH 7.2).

### 4.8. CV Histochemistry

CV histochemical staining was performed to examine the distribution and morphology of cells in the hippocampus. In short, according to a published method [58], 1% CV acetate (Sigma, St. Louis, MO, USA) solution (in distilled water) was prepared, and glacial acetic acid was added to this solution. To stain the sections, they were reacted in the CV solution for 1 h at room temperature (about 23 °C). After washing the sections with distilled water, they were dehydrated with serial ethanol. Finally, the stained sections were prepared as permeant slides.

### 4.9. FJB Histofluorescence Staining

FJB is a fluorescent derivative used to detect degenerating cells. In this study, FJB histofluorescence staining was done to examine the damage/death of hippocampal cells after ischemia. As described previously [55,59,60], in short, the sections were immersed in a 0.06% solution of potassium permanganate and reacted with 0.0004% solution of FJB (Histochem, Jefferson, AR, USA).

### 4.10. Data Analysis

Changes in CB, Iba-1 and GFAP immunoreactivity were quantitatively analyzed according to our published method [38]. In brief, we selected seven sections at 120 μm intervals within the antero-posterior from −1.4 to −2.2 mm according to the gerbil brain atlas in each gerbil. Images of each immunoreactivity were taken from the corresponding area (250 µm^2^) under 20× primary magnification in the hippocampus with an AxioM1 light microscope from Carl Zeiss (Göttingen, Germany) equipped with a digital camera from Axiocam (Carl Zeiss, Germany), which was connected to a PC monitor. The captured images were calibrated into an array of 512 × 512 pixels, and each immunoreactivity was evaluated by optical density (OD). The OD was obtained after the transformation of the mean grey level using a formula (OD = log (256/mean grey level). The background density was subtracted, and the OD ratio was calibrated as the percent of relative OD (ROD) using Adobe Photoshop version 8.0. Finally, ROD was analyzed with the Image J 1.46 software from the National Institutes of Health (Bethesda, MD, USA).

Numbers of NeuN-and FJB-positive cells were counted according to our published method [61]. In brief, we selected seven sections via the above-mentioned method. Images of NeuN-positive cells were captured with an AxioM1 light microscope (Carl Zeiss, Göttingen, Germany). Images of FJB-positive cells were taken with an epifluorescent microscope from Carl Zeiss (Göttingen, Germany) equipped with 450–490 nm of blue excitation light and a barrier filter. The digital images of cells positive for NeuN and FJB were counted in a 250 × 250 μm square applied at the center of the CA1 region using an image analyzing software (Optimas 6.5) from CyberMetrics (Scottsdale, AZ, USA).

### 4.11. Statistical Analysis

Data are expressed as the mean±SEM (standard error of the mean). The differences in the ROD or mean numbers of the respective immunoreactive structures obtained for each group were statistically analyzed with one-way analysis of variance followed by a post hoc Tukey’s test using GraphPad Instat from Instat Statistics (GraphPad Software Inc., La Jolla, CA, USA). A *p* value of <0.05 was considered statistically significant.

## Data Availability

The data presented in this study are available on request from the corresponding author.

## Abbreviations

BBB	Blood–Brain Barrier
CA	Cornu Ammonis
CB	Calbindin D28K
CV	Cresyl Violet
FJB	Fluoro Jade B
GFAP	Glial fibrillary Acidic Protein
Iba-1	Ionized Calcium Binding Adapter Molecule 1
IF	Intermittent Fasting
ND	Normal Diet
NeuN	Neuronal Nuclear Antigen
PAT	Passive Avoidance Test
ROD	Relative Optical Density
SO	Stratum Oriens
SP	Stratum Pyramidale
SR	Stratum Radiatum
TFI	Transient Forebrain Ischemia
